# Opening Address of the Atlanta Medical College, Oct. 1st, 1890

**Published:** 1890-11

**Authors:** H. L. Jones


VOL. VII. NOVEMBER, 1890. No. 9.

### Original Communications.

### OPENING ADDRESS OF THE ATLANTA MEDICAL COLLEGE OCTOBER, 1ST, 1890.

BY PROF. H. L. JONES.

Man is very intimately related to the material world around him. The closeness of this relation is recognized and acknowledged by mankind in the familiar expression, "Old Mother Earth," and is beautifully and impressively emphasized in Grecian mythology by the story of the giant Antæsos. Hercules, the type of strength, attacked, and long tried his strength upon him, without success, for as often as he threw him on his Mother Earth, he rose with renewed force and spirit. Hercules, finding his strength vain and his efforts futile, at length lifted Antæsos, in his mighty arms, above the earth, and holding him in mid-air, the strength of Antæsos departed and Hercules at once overpowered and destroyed him.

The correctness of these general and imaginative conceptions of mankind have been fully confirmed by scientific investigation. Man is the offspring of the earth; his body is composed of materials furnished by it, and his mind acquires its development largely, to say the least, from the impressions the material world makes upon it through the senses. However theologians and scientists may differ as to the precise manner in which man originated—whether he came full-formed and direct from the hands of his Maker, or indirectly, by derivative creation, through long descent from monad, mollusk, fish, reptile, mammal, ape and savage, to his present high estate; whichever, I say, of these hypotheses be true, chemistry places beyond doubt the fact that his body is made up wholly and entirely of constituents that are found in earth, water and air, and that there is no essential difference between the elements of his body and those of plants, birds and beasts; yea, even of the ground itself. That man was made of the dust of the earth, science teaches no less than revelation. Science tells us that a portion of the general stock of matter belonging to the earth is constantly entering into his body in the shape of food; that it becomes assimilated and enters into the constitution of his bones, muscles, nerves and brain, and that another portion, which had previously entered, is as constantly passing out through its various excretions. In the popular mind, all the matter of the body is supposed, by this inflow and outflow, to be completely changed every seven years. How this idea originated we do not know, unless it was suggested by the mystic number 7, which the imagination of mankind has connected with so many important things. Experiments, however, have shown that some of the matters taken into the body escape through the excretions in a very few hours, and it is altogether probable that some of the matter in the human body is removed and replaced in less than twenty-four hours, whilst some may remain for a lifetime. The constituents of muscles and nerves are being incessantly renewed, whilst those of bone and cartilage are very permanent.

Now, it is important for us to note that the matter thus withdrawn from the general stock for a little while, and incorporated in the tissues of the human body, does not, in consequence of this transfer, lose its distinctive qualities. Carbon, hydrogen, oxygen, nitrogen, sulphur, calcium, etc., when they pass into the human body are carbon, hydrogen, oxygen, etc., still. They are placed, it is true, under different conditions, and establish, perhaps, new relations among themselves, but they still remain subject to the laws which previously controlled them. The human body is as much subject to the force of gravity as the stones beneath its feet. It can be burnt by fire; it can rot and decay. It receives, conducts and loses heat like other portions of matter; it can be electrified and transmit electric currents like other bodies. The laws that govern the motions of fluids in general, control the circulation of the blood; the pressure exerted by the heart is felt at the ends of the arteries. Osmotic action is ever causing absorption and excretion of fluids in stomach, intestines, glands and capillaries. Alkalies and fats form emulsions in the alimentary canal, just as they do in a shaken flask, and acids in the stomach dissolve albuminoids as readily as they do in retorts in the laboratory. Iron reddens the corpuscles of the blood, as it does the molecules of clay beneath our feet, and oxygen, uniting with the various substances of the body, generates heat just as it does in stove and furnace. In short, man's body, the materials of which it is composed, and the functions it performs, are, one and all, governed by natural forces, and are the subject of natural laws. Of all the lessons a physician is called upon to learn, this is the most fundamental and important, viz.: that everything is governed by law; that nothing happens by chance; that the laws impressed upon matter, whether living or dead, are as immutable as their Author.

Natural phenomena do not appear to the untutored savage, and to the more ignorant of mankind, to be under the dominion of law. The physical universe appears to them chaotic, disjointed and disconnected. A few phenomena only, such as succession of day and night, the alternations of the seasons, the changes of the moon, and the ebb and flow of the tides, to them seem wrested from the dominion of chaos and brought within the reign of law. But as the boundaries of knowledge are enlarged, especially scientific knowledge, an increasing number of phenomena are made the subjects of law and order. The discovery by Newton of the force of gravitation, coextensive with the visible universe, and governed everywhere by a law capable of the same mathematical expression, led the way to the demonstration that the sun controlled the motions of the earth and the associated planets, that not only the earth, as a whole, but all its component parts, solids, fluids and gases, are subject to its sway. Even the paths of the winds are being mapped out as the resultants of effects of heat, rotation of the earth and of gravity, as expressed in terms of pneumatic pressure. Heat and electricity, statical and galvanic, obey laws now well understood. Chemistry has shown that the burning of coal, the decay of wood, the putrefaction of animal bodies, are all essentially the same, and all the subjects of laws. Vital phenomena, no less than those of dead matter, are yearly shown to be more and more under the dominion of law. Insurance companies rely with confidence upon the probabilities of life in a given case; which simply means that death and life are governed by laws that may be formulated. Statistics show that births and deaths, also the relative number of the sexes, are subject to law ; though we may not yet be able to formulate their laws with entire precision. Indeed, so universally, and without exception, has an increase of knowledge brought a greater and greater number of phenomena of all kinds under the dominion of law, that we deem it neither rash nor unphilosophical to express the belief that everything in the universe is the subject of law—life, health and disease included.

We now advance one step further. Not only are all things under the dominion of law, but there are powers or forces behind these laws amply able to enforce and execute them. Man may build daws across streams and temporarily check the flow of water, but gravity will eventually carry it to the sea. Our first duty, then, is to become acquainted with these laws; our second is to obey them. The sciences, so far as they are developed and perfected, are simply formulations of these laws, and as man touches nature on every side, and is, in turn, acted upon by her, the physician should embrace in his grasp a knowledge of the sciences, especially physics and chemistry. The name *physician* comes from the word *physics*, which in earlier times was equivalent to all the natural sciences. Physicians then were presumed to be students of nature in the broadest sense, and not mere dispensers of drugs. When a physician limits his studies to the cure of disease, neglecting to go deeper and seek for causes; to interpret the laws of nature and apply them; he then works empirically; he forfeits his right to the name physician, and becomes what the Germans call a "mediciner," or the Indians, a "medicine man." His business is to regulate the workings of the human organism, to ward off disturbances, and to right the machinery when thrown out of gear. Can he do this intelligently and successfully if he is ignorant of the forces operative upon and in this organism, and of the laws that direct and control these forces? Surely a thorough education in physics and chemistry is of the very first importance to a physician, and if practicable, should precede matriculation in the medical college proper. It cannot be impressed upon you, gentlemen, too deeply, that a knowledge of physics and chemistry is essential to a well equipped physician, and that medical colleges have failed to stress these in their curricula, not because they do not deem them important, but because other institutions of learning afford facilities for acquiring a knowledge of them, and because the rush and hurry of the times compel the medical colleges to curtail their curricula as much as possible.

Our second point is, that after acquiring a knowledge of the laws of nature, our highest duty is to obey them. Not only so, but our greatest good demands that we do it. It is idle for men to contend against the forces of nature. All realize this in the grand manifestations of the tempest, the thunderbolt, the surging waves and the earthquake shock. But it is none the less true in the case of those quiet, unseen, inaudible forces, which, like the expansion of freezing water, rends the solid rocks. Whilst we cannot successfully contend against the forces of nature, we may direct them into channels where their workings may be useful and promotive of our good. We may not be able to stop the water of a stream, as it hurries onward to the ocean, but we may turn it aside into new channels and make it drive the wheels of factories and mills, and furnish the power for converting nature's products into fabrics useful for our comfrot and adornment. We can but bow and tremble before the whirling cyclone, but we may utilize the gentle breeze to fill the white sails and speed onward the commerce of the world. By conforming to the laws that govern the forces of nature, we may utilize these forces and make them subservient to our good.

The wonderful progress of all the arts and industries in modern times is due to the development of science, the discovery of more of nature's laws, and the directing of the forces acting under these laws, into useful channels. When we conform to these laws, the forces behind them are most excellent handmaids; when we oppose and resist them, they are transformed into mailed warriors, armed with weapons of destruction.

But enough of these general considerations. I hope I have not failed entirely in impressing upon you the importance of a knowledge of the sciences as the basis of a medical education. It is proper, perhaps, on this occasion, to emphasize more specifically the claims of chemistry in this connection.

Let me remind you in the first place, that the treatment of disease involves vastly more than the mere administration of drugs. Among other things, your patient must eat and drink, and the proper regulation of diet often ranks first in importance in the treatment of a case. To decide what kind of food should be eaten and what should not, and how such food is to be prepared, you must know its composition, the chemical changes involved in its preparation, the composition of baking powders, the reaction of buttermilk and soda; the relations of food to muscular power, to animal heat; the digestibility and indigestibility of different foods; and such knowledge can come only from an acquaintance with chemistry. Without such knowledge, your advice in a given case can neither be intelligent nor trustworthy, satisfactory to yourself nor beneficial to your patient. Grandmothers and maiden aunts will often oppose you with all the positivism and assertion of ignorance, and to stand firmly, you must know the ground upon which you stand. *Doubt*, no less than conscience, makes cowards of us all.

Digestion is almost purely a chemical process, susceptible of imitation outside of the living body. The digestibility of foods can be determined in the laboratory almost as well as it was by Beaumont in the stomach of Alexis St. Martin, by experiment and observation. The results in his case were vitiated by personal idiosyncrasy; in laboratory experiments this is eliminated. Again, the disposition of wines, beers, etc., to ferment in the stomach, depend on the nature and extent of the chemical changes undergone by the substances of which they were formed. The effects they produce on the system, vary with the percentage of alcohol, of carbon dioxide and the various acids or ethers, present in them. Can any one, without a knowledge of chemistry, fully understand and appreciate these things. Much of course may be learned from ordinary experience and observation; something may be learned from tables in chemical books, giving percentage, compositions of foods and drinks, but we hesitate not to say, that persons not versed in chemistry are not prepared and cannot appreciate the full meaning of such tables. Experience and observation must be interpreted; a dozen different conclusions may be drawn from the same experiences and observa– tions, according to the knowledge of the observer and the standpoint from which he views them. Is it unreasonable to assert that a physician who is familiar with chemistry is better prepared to interpret aright experiences and observations in which chemical reactions are involved, than one who has no knowledge of chemistry?

You will, frequently, also be consulted concerning mineral waters. As a rule mankind have great faith in these. In not a few instances their curative effect is a matter of faith truly and emphatically; in others, their curative power in some diseases is real and undoubted. Concerning these you ought to be in a position to advise intelligently. Mineral waters hold chemical compounds in solution; some hold them for a little while only after they issue from the earth and are exposed to the air; some hold them in permanent solution; some, therefore, will bear bottling and shipping, others will not. A knowledge of chemistry will acquaint you with these differences, and enable you to recommend this or that rationally—not blindly and at hap-hazard. Ability to discuss these matters intelligently will commend you to your patients, increase their faith in the waters and thus enhance their curative power. In one sense *faith cures* are quite as good as any others.

You will be consulted not only touching mineral water, but about the purity and impurity of drinking waters and their relations to disease. Those of you who may chance to live in cities of any size, will find this a matter of first and pressing importance, and the family physician will be looked to for guidance and direction in the premises. To what other source than chemistry can you look for your own guidance in forming an opinion? Without a knowledge of chemistry you would, in these matters, stand upon the same footing, exactly, as your patients.

So also in cases of poisoning, where life is trembling in the balance, when decision must be quick and action prompt, how helpless you would feel without a knowledge of chemistry. But your responsibility may not end in the treatment of poisoned persons. You may be summoned to appear in court as an expert witness; if ignorant of chemistry, how easy may it be for a sharp lawyer to expose your ignorance and destroy your infallibility. Ignorant in one, ignorant in all, may, and probably will, be the popular verdict.

You will be looked to as authority by your patients, in questions touching the selection of climates for invalids. You must decide whether the consumptive shall go to the mountains or the seashore; what change of climate will best suit the chronic case that has baffled all your therapeutic skill. This opens up the question of the relation of atmospheric conditions to disease; why one locality is infested with an epidemic and a similar and may be adjacent one escapes. Here is a field for study broad enough to satisfy the ambition of an Alexander, and for the solution of which you will need all your scientific knowledge and skill.

In administering drugs you will have frequent occasion to give a number of them at the same time. It often happens that, whilst the therapeutic effects of the several ingredients in a prescription may be harmonious and desirable, the chemical reactions among them, when mixed together, may be anything else than harmonious. In medical language, they may be entirely incompatible with each other. A physician unacquainted with chemistry is ever liable to make serious and ludicrous mistakes in his prescriptions. To memorize and hold in memory the names and qualities and incompatibilities of all the substances in the pharmacopœia is more than can be expected of mortal man, but the knowledge of a few fundamental facts and principles of chemistry, within easy reach and grasp, will guide a physician safely through them all.

And lastly, young gentlemen, remember, as an ambition worthy of you all, that the death rate of the world, the longevity and happiness of mankind is, to a large extent, in the hands of each and every one of you, and whether the mortality shall increase or decrease in the localities where you practice will depend upon how thoroughly you have mastered the laws of nature, and how dutifully and skilfully you apply them.

Strive, by benefiting humanity to make for yourselves names that shall go down to posterity with those of Marion Sims, Sam'l Gross, Chas. D. Meigs and your own Westmoreland.

